# Effects of a 24/48 to 48/96 Shift Schedule Change on Firefighter Sleep and Health: Short-Term Improvements and Six-Month Stability

**DOI:** 10.3390/ijerph22111678

**Published:** 2025-11-05

**Authors:** Joel M. Billings, Sara A. Jahnke

**Affiliations:** 1 Department of Emergency, Disaster and Global Security Studies, Embry-Riddle Aeronautical University, Daytona Beach, FL 32114, USA; 2 Center for Fire, Rescue & EMS Health Research, NDRI-USA, Leawood, KS 66224, USA; jahnke@ndri-usa.org

**Keywords:** firefighter sleep, 24/48, 48/96, shift schedule, call volume

## Abstract

This study examined the impact of a shift schedule change on firefighter sleep and health outcomes (*n* = 24). Firefighters from a U.S. department transitioned from a 24 h on, 48 h off (24/48) schedule to a 48 h on, 96 h off (48/96) schedule. Wrist actigraphy and self-reported health outcomes were assessed at three time points: baseline (24/48), 3 months post-transition, and 6 months post-transition. Objective sleep measures included total sleep time (TST), sleep efficiency (SE), sleep onset latency (SOL), and wake after sleep onset (WASO). Self-reported health outcomes included the Insomnia Severity Index (ISI), Beck Depression Inventory–II (BDI-II), Beck Anxiety Inventory (BAI), Multidimensional Assessment of Fatigue (MAF), and the Alcohol Use Disorders Identification Test (AUDIT). Linear mixed-effects models (LMMs) with random intercepts were used to evaluate changes over time, adjusting for age, years of service, and individual night-time call volume. Results showed significant improvements in TST, SE, SOL, and WASO at the 3-month follow-up, which were sustained but did not further increase at 6 months. ISI and BDI-II scores also improved, while BAI, MAF, and AUDIT remained stable. These findings suggest that the 48/96 schedule may provide short-term improvements in sleep and psychological health for firefighters in low call-volume settings. Additional research is needed in higher-volume departments and over longer timeframes.

## 1. Introduction

The purpose of the research reported herein is to examine sleep and health over time among firefighters in a fire department that changed shift schedules. Sleep is a foundational necessity for physical, cognitive, and emotional functioning [1]. Yet, for certain populations, particularly shift workers, obtaining sufficient and restorative sleep is often difficult due to working irregular schedules that can cause circadian misalignment [2]. Among shift workers, fire and emergency service personnel experience particularly severe challenges on obtaining sufficient sleep at work due to remaining in a constant state of operational readiness [3], frequently waking by loud emergency alarms, which have been shown to raise heart rates to over 100 bpm within seconds of waking [4,5], and responding to emergency calls of various and unpredictable workload and durations [6,7,8]. These conditions impact sleep opportunities and sleep quality at work. In fact, recent research using EEG-based, at-home polysomnography has demonstrated that firefighters experience significantly clinically meaningful differences in stages of sleep when sleeping at the station, in addition to greater cortical and autonomic arousals and reduced spindle activity compared to sleeping at home [9]. Even at home, sleep can be impacted due to firefighters prioritizing childcare and household responsibilities over sleep [10]. Therefore, the repeated disruptions at work and questionable recovery at home can place firefighters in a repeat cycle of insufficient sleep and incomplete recovery across days within their tour [11].

Studies of firefighters show that sleep deprivation impairs simple reaction time, vigilance, and processing speed, leading to more cognitive errors and slower responses during duty [12,13]. Recent research has further linked occupational stress and shift work to reduced cognitive performance among firefighters, highlighting the combined impact of disrupted sleep and job-related stress on safety and operational effectiveness [14]. When sustained over time, this chronic pattern may produce physiological strain that compounds across time [15,16,17] increasing vulnerability to adverse human health consequences. These can include cardiovascular disease [17,18], depression [19], cognitive impairment [12], obesity [20], and elevated injury risk [21]. Considering firefighter already face elevated physiological and psychological demands [22], these risks not only affect individual firefighters, but may also impair emergency response performance and increase the potential for adverse incidents including motor vehicle accidents [21]. Therefore, firefighter sleep health represents not only an occupational health priority but also a broader public health concern, with implications for responder safety, community protection, and the sustainability of fire service operations.

Fire departments in the United States typically operate using 24 h shift schedules to ensure continuous emergency service coverage [23]. While this structure supports operational readiness, such irregular structure of 24 h on- and off-shifts often conflicts with the body’s biological need for sustained sleep and recovery [16]. Although firefighters may have opportunities to rest during shifts when not actively responding to calls, sleep is not guaranteed particularly among firefighters who experience frequent night-time emergency calls [6,7]. This causes unpredictability and inconsistency in sleep opportunities and as a result, has prompted some fire departments to reevaluate their shift schedules in an effort to promote firefighter health, performance, and overall well-being.

In the broader workforce, conversations about scheduling intensified during the COVID-19 pandemic, with some organizations adopting flexible arrangements or giving employees greater control over their schedules [24,25]. Such approaches have been shown to improve sleep and work–life balance in certain occupations [26]. In the fire service, however, staffing requirements for continuous emergency coverage limit such options, and schedules are largely fixed at the departmental level. Unlike other occupations that may offer nearly unlimited flexibility in when and where work is performed, flexibility in the fire service is largely confined to the pattern of work and non-work days (i.e., shift schedule) within the designated work period.

One increasingly popular change is the transition from the traditional 24 h on/48 h off (24/48) shift schedule to the 48 h on/96 h off (48/96) schedule. Both schedules use three platoons (crews/shifts), that rotate shifts for continual coverage. The 24/48 operates on a 3-day tour whereas the 48/96 operates on a 6-day tour. While the 24/48 shift schedule is the most common [23], some evidence suggests that because the tours cycle quickly, it may not provide adequate recovery time between shifts, especially when the final night’s sleep at home is truncated by early wake-ups to report for duty [11]. In contrast, the 48/96 schedule consolidates two 24 h shifts but offers an extended recovery period at home, which could promote better sleep and improve work–life balance [27], depending on workload. However, questions remain about whether working two consecutive 24 h shifts may contribute to cumulative fatigue or disrupt sleep patterns, particularly among firefighters in high call volume stations.

Research examining how these shift schedules affect firefighters’ sleep has yielded mixed findings, and longitudinal studies remain limited. Some research indicates no statistical differences in sleep quality between the 24/48 and 48/96 [28], while others have reported more REM sleep on the 48/96 compared to the 24/48 [29]. In a study that assessed the 48/96 and Kelly (i.e., 24 h on, 24 h off, 24 h on, 24 h off, 24 h on, 96 h off) shift schedules, researchers found improvements in sleep duration, burnout, and daytime sleepiness on the 48/96 [30]. Yet most prior work has examined schedules in isolation, with little attention to how workload (e.g., call volume) interacts with schedule structure to study sleep outcomes both on and off duty. In addition, few studies have followed firefighters across multiple post-transition intervals, leaving limited understanding of how quickly benefits emerge after a schedule change and whether evolve over time.

To address these gaps, the present research evaluates longitudinal sleep data among firefighters who transitioned from a 24/48 to a 48/96 shift schedule. Although prior analyses from this cohort have documented sleep patterns six months after the schedule change [11], this study introduces new findings by examining data collected at three months post-transition, providing a clearer picture of the trajectory of sleep outcomes across time. We focus on key objective sleep parameters: total sleep time (TST), sleep efficiency (SE), sleep latency (SL), and wake after sleep onset (WASO) across these three periods. The findings aim to inform departmental scheduling decisions, guide occupational health strategies, and refine research protocols by highlighting the importance of accounting for workload timing intervals in sleep assessments following shift schedule transitions.

## 2. Materials and Methods

### 2.1. Study Design

This study used a pre-experimental, within-subjects, longitudinal design to evaluate changes in firefighters’ sleep associated with a shift schedule transition. Data collection occurred at three time points: (1) baseline (24/48), (2) three months post transition (48/96), and (3) six months post transition (48/96).

### 2.2. Fire Department and Participants

Participants were recruited from a career fire department in the Southcentral United States. The department employed 64 firefighters across four stations, responded to approximately 5000 calls annually, served a community of 50,000 residents, and began and ended shifts at 7:00 AM. All 64 firefighters were approached in person during station visits while on duty. During these visits, the PI provided a brief overview of study objectives and procedures and then invited firefighters to ask questions and enroll individually. Participants were required to be full-time firefighters with at least two months of current continuous work experience at that department under the current shift schedule, which helped ensure their circadian rhythms not aligned to any previous work schedule.

**Figure 1:**
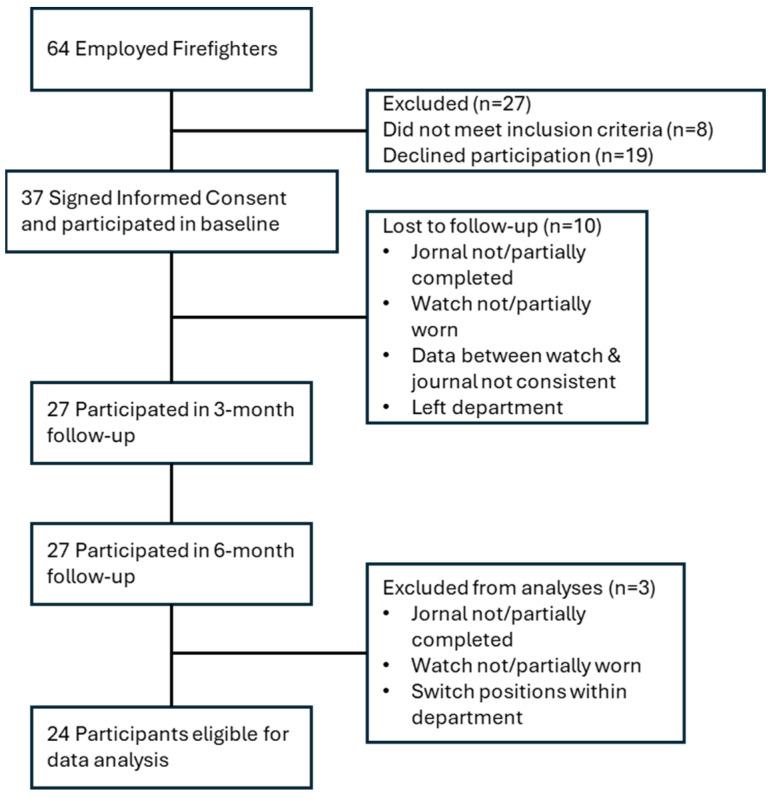
Participant Flow.

Thirty-seven (58%) firefighters provided written informed consent to participate (approved by Oklahoma State University’s Institutional Review Board). Twenty-seven were excluded prior to baseline, including 19 who declined participation and 8 who did not meet inclusion criteria. Ten consented participants did not complete required baseline elements and were removed per protocol, leaving 27 who completed the three-month and six-month follow-ups. Three of these were subsequently excluded due to protocol deviations or a position change within the department, yielding a final analytic sample of 24. Participant flow is illustrated in Figure 1 and baseline characteristics in Table 1. For more details on sample characteristics, refer to previous published research [11].

### 2.3. Sleep Measures

Objective sleep measures were collected using the Actigraph wGT3X-BT device, worn continuously on the non-dominant wrist. While in-lab polysomnography (PSG) is considered the gold standard for characterizing sleep, its use was not feasible in this study due to resource limitations. Although PSG has recently been deployed successfully in the firefighter population [9] wrist actigraphy remains a validated, less intrusive, and widely used method for measuring sleep duration [31,32]. Actigraphy assessment provides objective sleep parameters such as TST (the total duration of sleep obtained during each sleep period), SE (the ratio of total sleep time to the total amount of time spent in bed, expressed as a percentage), SOL (the duration between initiating an attempt to sleep and the onset of sleep), and WASO (the total duration of wakefulness occurring after initial sleep onset) for each sleep episode.

In addition to wearing wrist actigraphy, participants were instructed to record their in-bed and out-of-bed times in a diary to minimize error and improve scoring accuracy of actigraphy data, as recommended for actigraphy assessment [33]. Participants used the Emergency Services Sleep Diary (ESSD) [34], designed specifically for fire and emergency service personnel, a population who experiences multiple sleep episodes during the night.

### 2.4. Heath Outcome Measures

Participants completed several validated, self-reported questionnaires at the beginning of each data collection round to evaluate health-related outcomes in association with the shift schedule change. These measures assessed various dimensions of health, including sleep difficulties, mental health, alcohol use, and fatigue.

Insomnia Severity Index (ISI): The ISI is a validated 7-item self-report instrument that assesses the perceived severity of insomnia symptoms over the past two weeks [35]. Participants rate the difficulty initiating and maintaining sleep, early morning awakenings, satisfaction with current sleep, and the impact of sleep problems on daytime functioning. Higher scores indicate greater insomnia severity.

Beck Depression Inventory-II (BDI-II): Depressive symptoms were evaluated using the BDI-II, a widely used 21-item questionnaire that measures the severity of depressive symptoms experienced during the previous two weeks [36]. Scores range from minimal to severe depression, with higher scores indicating more severe depressive symptoms.

Beck Anxiety Inventory (BAI): Anxiety symptoms were measured using the BAI, a validated 21-item questionnaire assessing common physical and cognitive symptoms of anxiety experienced over the past week [37]. Scores range from minimal to severe anxiety, with higher scores reflecting greater anxiety severity.

Alcohol Use Disorders Identification Test (AUDIT): Participants’ alcohol consumption behaviors and related risks were assessed using the AUDIT [38]. This 10-item self-report instrument evaluates alcohol use frequency, quantity, dependence symptoms, and harmful alcohol use patterns over the past year. Higher scores suggest increased risk for hazardous or harmful drinking.

Multidimensional Assessment of Fatigue (MAF): Fatigue was assessed with the MAF, a validated self-report measure evaluating multiple dimensions of fatigue, including severity, distress, degree of interference with daily activities, and timing [39]. Higher scores indicate greater fatigue severity and impact.

### 2.5. Data Collection Protocol

During baseline, 3-month follow-up, and 6-month follow-up, participants completed a demographic and health questionnaire, were administered wrist-worn actigraphy devices, and were provided instructions and training on the completion of the sleep diary each morning, afternoon, and evening throughout the 18-day periods.

Actigraphy guidance recommends extended continuous recording (≥7 days) in naturalistic environments, nondominant wrist placement, and concurrent use of sleep diaries to improve scoring accuracy [33]. Therefore, firefighters were asked to wear the actigraphy device continuously for 18 days for each data collection period on nondominant wrist, and complete the ESSD. The 18-day periods provided ample tour data (six tours worth of data under the 24/48 and three tours of data under the 48/96, for each period) in case participants deviated from the schedule (e.g., sick leave, vacation leave, overtime, swapping shifts, etc.). This data would be excluded from analysis since it would represent a different work shift.

### 2.6. Data Preparation

Actigraphy data were processed using ActiLife, version 6.13.6. Data were first validated for non-wear using the Choi and colleagues [40] algorithm in ActiLife. The sleep and wake times recorded in the ESSD were manually entered into ActiLife to define each sleep period, which was then was scored using the Cole-Kripke [41] algorithm. and exported into CSV files for further processing. When participants experienced multiple sleep episodes during a single night (e.g., due to emergency responses), the sleep parameters from each episode were combined to create one nightly set of sleep parameters per participant. Eligible tours, defined as the set of consecutive nights in which a participant followed a cycle of the shift schedule) were then averaged so that each participant had an overall mean value of sleep parameters.

Tours in which participants deviated from their assigned schedule were excluded to avoid mischaracterizing sleep patterns, as deviations alter the recovery periods inherent to the 24/48 and 48/96 schedules. Deviations were recorded at each time point, including 42 exclusions at baseline, nine at the three-month follow-up, and 12 at the six-month follow-up. The greater number of deviations during the 24/48 assessment likely reflects three factors: (1) twice as many tours were monitored in an 18-day period, creating more chances for deviation; (2) data collection occurred over the winter holiday season, when leave and shift trades are more common; and (3) it is easier to take leave on a 24 h shift than on a 48 h shift, since partial coverage of a longer shift can be more difficult to arrange.

### 2.7. Data Analysis

Data were analyzed using linear mixed-effects models (LMMs) with random intercepts for participants to account for repeated measures. Models included age, years of service, and individual night-time call volume as covariates. Interaction terms between time and call volume were also tested. LMMs were selected because they appropriately model correlated repeated measures data and accommodate potential imbalance in the number of observations per participant.

## 3. Results

### 3.1. Sleep Outcomes

LMM analyses indicated significant changes in TST across time points (Wald χ^2^(2) = 14.0, *p* = 0.015). Adjusted means increased from 383 min at baseline to 406 min at 3 months and 398 min at 6 months. Pairwise comparisons indicated that TST was significantly greater at both 3 months (+23 min, 95% CI 8.7–38.0, *p* = 0.002) and 6 months (+15 min, 95% CI 0.25–29.7, *p* = 0.046) compared with baseline, whereas the difference between 3 and 6 months was not significant (−8 min, *p* = 0.27). Night-time call volume was associated with shorter sleep duration (−15 min per call, 95% CI −30.3 to 0.8, *p* = 0.063), although this effect did not reach significance. Interaction terms were not significant, indicating that schedule-related improvements in sleep were consistent across call volumes. Both schedule structure and call volume exerted independent effects.

A strong effect of time was observed for SE (Wald χ^2^(2) = 23.1, *p* < 0.001). SE increased from 84.6% at baseline to 88.5% at 3 months (+3.9%, 95% CI 2.0–5.9, *p* < 0.001) and 87.8% at 6 months (+3.2%, 95% CI 1.2–5.2, *p* = 0.002), with no difference between 3 and 6 months (−0.7%, *p* = 0.47). Call volume was associated with lower efficiency (−1.4% per call, *p* = 0.19). Interaction terms between time and call volume were not significant, indicating that schedule-related improvements in SE were consistent across call volumes.

SOL decreased significantly across time (Wald χ^2^(2) = 18.3, *p* = 0.003), from 8.7 min at baseline to 4.9 min at 3 months and 4.1 min at 6 months. Reductions were significant at both 3 months (−3.9 min, 95% CI −6.4 to −1.3, *p* = 0.003) and 6 months (−4.6 min, 95% CI −7.2 to −2.1, *p* < 0.001), with no further change from 3 to 6 months (−0.8 min, *p* = 0.55). Neither call volume nor demographic covariates were significant predictors.

**Figure 2:**
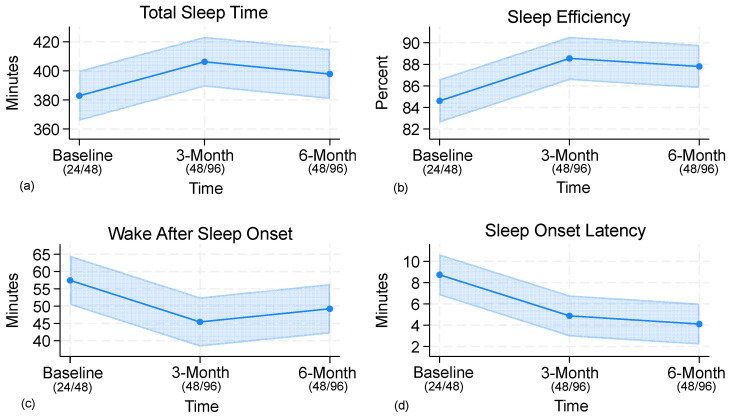
Trajectories of sleep outcomes: (**a**) total sleep time (TST), (**b**) sleep efficiency (SE), (**c**) wake after sleep onset (WASO), and (**d**) sleep onset latency (SOL) across baseline (24/48), 3 months post-transition (48/96), and 6 months post-transition (48/96). Lines represent adjusted means from LMMs, and shaded areas represent 95% confidence intervals.

WASO also decreased following the schedule change (Wald χ^2^(2) = 19.3, *p* = 0.002). Adjusted means declined from 57.5 min at baseline to 45.4 min at 3 months (−12.1 min, 95% CI −19.1 to −5.0, *p* = 0.001) and 49.2 min at 6 months (−8.2 min, 95% CI −15.4 to −1.1, *p* = 0.023). No difference was found between 3 and 6 months (+3.8 min, *p* = 0.29). Years of service showed a small positive association (+1.2 min per year, *p* = 0.035), while call volume did not significantly predict WASO. Interaction terms were not significant, indicating that the pattern of WASO improvement over time was consistent across call volumes. Adjusted means and pairwise comparisons are presented in Table 2, with raw means provided in Table 3, and Figure 2 illustrates trajectories of sleep outcomes.

### 3.2. Health Outcomes

Depressive symptoms (BDI-II) decreased significantly over time (Wald χ^2^(2) = 23.1, *p* < 0.001), from 5.3 at baseline to 3.9 at 3 months and 3.3 at 6 months. Reductions were significant at both 3 months (−1.4 points, 95% CI −2.4 to −0.5, *p* = 0.004) and 6 months (−2.0 points, 95% CI −3.0 to −1.0, *p* < 0.001), with no significant change between 3 and 6 months (−0.6, *p* = 0.25). Years of service was associated with higher BDI-II (+0.34 per year, *p* = 0.028). Night calls showed a negative association (−0.43 per call, *p* = 0.049).

Reductions in ISI scores were observed but the overall effect did not reach statistical significance (Wald χ^2^(2) = 9.7, *p* = 0.083). Mean ISI decreased from 7.4 at baseline to 6.5 at 3 months and 5.5 at 6 months. The baseline-to-6-months contrast was significant (−1.9 points, 95% CI −3.1 to −0.6, *p* = 0.003), whereas baseline-to-3 month (−0.9 points, *p* = 0.17) and 3-to-6 month differences (−1.0 points, *p* = 0.12) were not. No significant covariate effects emerged.

No significant changes were observed in anxiety symptoms (BAI) (Wald χ^2^(2) = 5.1, *p* = 0.41), alcohol use (AUDIT) (Wald χ^2^(2) = 0.17, *p* = 0.92), or fatigue (MAF) (Wald χ^2^(2) = 7.5, *p* = 0.19). Adjusted means for these outcomes remained stable across baseline, 3-month, and 6-month assessments, and pairwise contrasts demonstrated no significant differences between rounds (all *p* > 0.05). Covariates including age, years of service, and call volume were not significantly associated with these measures.

### 3.3. Covariates

Age was not associated with any sleep or health measures. Years of service predicted higher WASO and BDI-II scores, while call volume showed consistent associations with poorer TST and SE, though effects did not consistently reach statistical significance. Importantly, time × call volume interactions were not significant in any model, indicating that schedule-related improvements were independent of call volume. This suggests additive rather than multiplicative influences of organizational scheduling and workload on firefighter sleep and health.

## 4. Discussion

The purpose of this research was to assess firefighter sleep and health following a department-wide transition from a 24/48 to a 48/96 schedule. The findings suggest short-term improvements across multiple outcomes. Using a within-subjects design, the data suggest that firefighters obtained significantly more TST and demonstrated greater SE under the 48/96 schedule. Sleep continuity also improved, as indicated by shorter SOL and less WASO. These gains in sleep were evident in the three-month follow-up and sustained at six months, although they did not continue to increase. Insomnia and depressive symptoms declined over time, while anxiety, fatigue, and alcohol use remained unchanged. Importantly, these changes persisted after adjusting for call volume, years of service, and age. This suggests that improvements were not limited to particular demographic subgroups or workload levels. Although years of service showed modest associations with greater WASO and higher BDI-II scores, and higher call volume was linked with less TST and lower SE, the absence of significant interactions suggests that schedule-related benefits were consistent across the firefighter sample.

Firefighters slept approximately 23 additional minutes of sleep and 3–4% higher efficiency per tour after then transition. A possible explanation is that the 48/96 schedule offered firefighters more flexibility in when to complete station duties. Under the 24/48 schedule, tasks had to be finished before the oncoming crew arrived, often requiring earlier wake times. In contrast, remaining at the station overnight during the 48/96 allowed certain responsibilities to be postponed until later in the day. This adjustment reduced the need for early awakenings, delayed sleep offset, and likely contributed to the observed improvements in both TST and SE. Prior research has shown that even small extensions of nightly sleep reduce cumulative sleep debt and improved alertness [42], which may translate to lower risk of fatigue-related errors. Since many fire department shift schedules are quickly rotating, with multiple tours each week, the cumulative effect of small nightly improvements becomes significant. It is therefore possible that the observed gains in our study are likely to compound across time, representing a meaningful improvement in long-term recovery and health even if absolute effect sizes appear modest. Further longitudinal research is needed to confirm this hypothesis.

An alternative explanation for the improvements in TST and SE is that the 48 h shift itself was so physiologically demanding that firefighters were simply more fatigued, leading to longer and deeper sleep during recovery days. Two indicators argue against this interpretation. First, individual call volume remained stable across the transition from the 24/48 to the 48/96, and average nighttime workload was relatively low, with most firefighters experiencing only one call per night. Second, no increases were observed in MAF or BAI scores, which would be expected to deteriorate if longer shifts consistently overwhelmed firefighters. Instead, the pattern of results suggests that the 48/96 created conditions more conducive to restorative sleep by reducing early awakenings tied to commuting, allowing later wake times overnight on shift, and supporting more regular sleep timing during the extended off-duty period. Yet, prior work in this department found greater sleepiness on the first afternoon at home during 48/96 compared to the 24/48 [43]. This raises the possibility of fatigue-related risks at certain points in the cycle, a concern reinforced by laboratory evidence showing that even moderate, sustained sleep restriction (e.g., 6 h per night) produces cumulative neurobehavioral deficits and increased lapses in alertness [44]. It is also possible that the four-day period provided sufficient recovery to restore alertness prior to starting a new tour. Departments should carefully weigh these risks when evaluating schedule changes.

The off-shift period of the 48/96 also appears to have supported broader recovery. The four consecutive off-duty days likely enabled more consolidated rest and more stable sleep–wake patterns. Extended recovery periods are important in shift-working populations because they allow for repayment of accumulated sleep debt and partial normalization of circadian rhythms [45]. Although full circadian realignment is unlikely given firefighters’ irregular light exposure and operational demands, the 48/96 schedule creates a longer recovery period for extended morning sleep [11]. By contrast, the 24/48 may provide insufficient recovery since the second off day is often truncated by early waking to commute back to the station. In practice, this means that across the three-day tour, firefighters typically have only one morning at home to sleep in without interruption assuming family, social, or environmental demands do not interfere. Across the six-day tour of the 48/96 schedule, however, firefighters have the potential for three uninterrupted mornings at home, and depending on departmental policies, possibly a fourth morning while still on shift, providing more consistent opportunities for recovery. These conditions likely contributed to the observed reductions in ISI scores and overall improvements in sleep, consistent with experimental evidence that prolonged nightly sleep enhances alertness, vigilance, and mood [46]. Moreover, if shift start/end times were delayed, aligning more closely with firefighters’ endogenous wake times, additional gains in sleep and health outcomes might be realized.

Beyond physiological recovery, longer consecutive off-shift periods may also reduce chronic stress and provide firefighters greater opportunities for family engagement and social recovery [10,47]. Under the 24/48 schedule, the quick rotations often meant that when firefighters returned home needing rest and recovery, it created conflict with their spouse and many firefighters reported neglecting their own sleep in order to meet family and childcare responsibilities [10]. In addition, our results showed reductions in BDI-II scores, suggesting that the benefits of extended recovery extend into psychological health. These findings align with the well-documented bidirectional association between sleep and mental health [48], and recent work in firefighters showing that sleep disturbances and mental health influence the risk of burnout [49]. However, some caution is warranted: baseline BDI-II scores were already low in this sample, limiting the potential for large changes. Nonetheless, even in the relatively low-symptom sample, the improvements suggest a potential value of schedule-related recovery opportunities for firefighter well-being.

An unexpected result was that higher nighttime call volume was associated with slightly lower BDI-II scores. This counterintuitive association was small and should be interpreted with caution. Because BDI-II reflects habitual symptoms over roughly two weeks while our call volume covariate included only night-time calls, the association may reflect residual confounding or measurement mismatch rather than a direct effect of night calls. Prior work generally links higher call loads with poorer mental health [7], which underscores this caution. As a tentative hypothesis, in a relatively low call volume setting, modest operational engagement could relate to better well-being. However, this possibility is speculative and warrants further study. More broadly, workload is an important contextual factor when considering schedule-related benefits. Prior research has shown that greater emergency call volume is associated with increased physical exertion and reduced sleep [6]. In our study, night-time call volume showed consistent, though not always significant, associations with shorter TST and lower SE. Thus, while the 48/96 shift structure may offer advantages under lower call volume conditions, the same benefits may not emerge, or may even be worse, in higher call volume departments where cumulative fatigue across 48 h tours is likely to increase without rest and recovery.

Another interesting finding is related to the assessment intervals. The extent of some sleep and health improvements did not continue beyond the 3-month post assessment. The lack of further statistically significant improvements from the 3-month to 6-month assessments may indicate that firefighters adapted relatively quickly to the new schedule, with the greatest sleep benefits realized early in the transition. Several explanations are possible. Initial improvements may reflect heightened attention to sleep following the schedule transition (a Hawthorne effect), seasonal variation across measurement periods, or a stabilization of sleep patterns at a new baseline. It is also plausible that firefighters initially allocated more off-duty time to recovery but gradually balanced additional sleep with competing family or secondary work responsibilities. Regardless of mechanism, the stabilization of effects highlights the importance of longitudinal follow-up in firefighter sleep research, as improvements may or may not continue to accumulate with time. This finding has implications for researchers designing firefighter sleep studies. It underscores the importance of carefully selecting follow-up intervals to capture notable changes in sleep, which was evident in the significant ISI improvements from baseline to the 6-month post, rather than 3-month post. It also supports further investigation into whether long-term follow-up periods would reveal maintenance, declines, or additional improvements in sleep and health outcomes.

The findings also have implications for department policy. The 48/96 schedule may provide short-term improvements in sleep and psychological health without requiring major staffing changes, since both the 24/48 and 48/96 maintain a three-platoon structure. However, due to the sample size, call volume, and assessment period reported herein, the benefits observed may not necessarily generalize to other departments or firefighters who experience higher call volumes or heavier workloads, where extended shifts coupled with greater calls could exacerbate fatigue.

Future research should address several important questions. First, studies should explore the mechanisms underlying the initial improvement in sleep and mental health outcomes. Additionally, longer-term follow-ups would clarify the trajectory of sleep and health outcomes beyond what was captured within this study and how these impact firefighters after retirement. Future investigations could also examine the influence of departmental policies or interventions that can impact sleep at work and at home. Finally, examining other schedules such as 24/72 (which also offers a longer recovery period compared to the 24/48) and departments with higher call volumes would provide greater knowledge to the field.

## 5. Limitations

This research has a few noteworthy limitations. While improvements in sleep parameters and some psychological health outcomes are promising, the relatively modest effect sizes suggest these changes, though statistically significant, might have limited practical significance in real-world contexts. Furthermore, gains plateaued by six months, raising questions about the durability of benefits. Further studies should explore individual differences or subgroup effects, for example, examining how firefighters at busier stations or those experiencing higher call volumes might respond differently to the 48/96 schedule.

Another limitation is the reliance on actigraphy rather than polysomnography (PSG), which has recently been shown to be feasible in firefighter populations. Actigraphy was selected due to the self-funded nature of this dissertation project, but future research with greater resources should incorporate PSG to validate findings and provide deeper insight into firefighter sleep. Although concerns about familiarization effects exist with more intrusive ambulatory EEG and blood pressure monitors [50], wrist actigraphy does not typically require acclimation beyond clear instructions and concurrent diary use [33]. In addition, the extended wear periods in this study further minimized any potential adjustment effects.

The sample size and possible self-selection bias may also limit generalizability. We did not assess individual chronotype or changes in diet, caffeine intake, or concurrent wellness initiatives, and seasonal variations that may have affected outcomes. While each firefighter served as their own control in the within-subjects design, which reduces intra-individual variability, multi-site studies with control groups will be needed to strengthen causal conclusions.

Lastly, many fire departments continue to use the 24/48 shift schedule, which limits the generalizability of these findings across the broader fire service. Extended shifts may also interact with contextual factors such as departmental culture, station routines, and family or childcare obligations, which could alter recovery opportunities.

## 6. Conclusions

This study found that transitioning from a 24/48 to a 48/96 shift schedule was associated with short-term improvements in firefighter sleep and select health outcomes. Improvement in total sleep time, sleep efficiency, and reductions in insomnia and depressive symptoms emerged by the 3-month post assessment and were maintained through the final, six-month post assessment. These benefits occurred without any observed deterioration in fatigue, anxiety, or alcohol use, which suggests that the 48/96 structure, in the context of low call volume, may help support sleep stability and recovery.

## Figures and Tables

**Table 1:** Demographic Characteristics.

Sex male, *n*	24
Average Age	34 (range 20–52)
Marital Status, *n*	19 (79%) married
With Children under 18, *n*	16 (67%) with children
Second Job, *n*	10 (42%) with second job
Years of Service	10.5 years
Average Duration from Home to Station	28 min
Average Wake Time to Shift Start Time	96.5 min
Average Individual call frequency at night	1.0 call
Average Individual call frequency during day	2.6 calls

**Table 2:** Adjusted Means and Pairwise Differences from Linear Mixed-Effects Models (LMM) on Sleep and Health Outcomes.

Outcome	Baseline (M)	3 Months (M)	6 Months (M)	Wald χ^2^(df)	*p*-Value	Baseline vs. 3 m Δ	Baseline vs. 6 m Δ	3 m vs. 6 m Δ
TST (min)	383	406	398	14.0 (2)	0.015	+23.3 (95% CI: 8.7–38.0), *p* = 0.002	+15.0 (95% CI: 0.25–29.7), *p* = 0.046	−8.3 (95% CI: −23.1 to 6.4), *p* = 0.27
SE (%)	84.6	88.5	87.8	23.1 (2)	<0.001	3.9 (95% CI: 2.0–5.9), *p* < 0.001	+3.2 (95% CI: 1.2–5.2), *p* = 0.002	−0.7 (95% CI: −2.7 to 1.3), *p* = 0.47
SOL (min)	8.7	4.9	4.1	18.3 (2)	0.003	−3.9 (95% CI: −6.4 to −1.3), *p* = 0.003	−4.6 (95% CI: −7.2 to −2.1), *p* < 0.001	−0.78 (95% CI: −3.4 to 1.8), *p* = 0.55
WASO (min)	57.5	45.4	49.2	19.3 (2)	0.002	−12.1 (95% CI: −19.1 to −5.0), *p* = 0.001	−8.2 (95% CI: −15.4 to −1.1), *p* = 0.023	+3.8 (95% CI: −3.3 to 10.9), *p* = 0.29
ISI	7.4	6.5	5.5	9.7 (2)	0.083	−0.9 (95% CI: −2.1 to 0.37), *p* = 0.17	−1.9 (95% CI: −3.1 to −0.63), *p* = 0.003	−1.0 (95% CI: −2.25 to 0.25), *p* = 0.12
BDI-II	5.3	3.9	3.3	23.1 (2)	<0.001	−1.4 (95% CI: −2.4 to −0.45), *p* = 0.004	−2.0 (95% CI: −3.0 to −1.0), *p* < 0.001	−0.58 (95% CI: −1.55 to 0.41), *p* = 0.25
BAI	2.5	2.2	2.1	5.1 (2)	0.41	−0.3 (95% CI: −1.3 to 0.64), *p* = 0.51	−0.5 (95% CI: −1.4 to 0.50), *p* = 0.34	−0.14 (95% CI: −1.1 to 0.82), *p* = 0.77
AUDIT	3.8	3.9	4.0	0.17 (2)	0.92	+0.1 (95% CI: −0.54 to 0.72), *p* = 0.78	+0.2 (95% CI: −0.48 to 0.80), *p* = 0.63	+0.07 (95% CI: −0.57 to 0.70), *p* = 0.83
MAF	18.2	15.9	16.4	7.5 (2)	0.19	−2.4 (95% CI: −5.1 to 0.31), *p* = 0.083	−1.9 (95% CI: −4.6 to 0.81), *p* = 0.17	+0.49 (95% CI: −2.2 to 3.18), *p* = 0.72

Adjusted means (M) are shown for baseline, 3 months, and 6 months. Pairwise differences (Δ) are estimated from LMM with age, years of service, and night-time call volume as covariates. TST = Total Sleep Time; SE = Sleep Efficiency; SOL = Sleep Onset Latency; WASO = Wake After Sleep Onset; ISI = Insomnia Severity Index; MAF = Multidimensional Assessment of Fatigue; BDI-II = Beck Depression Inventory-II; BAI = Beck Anxiety Inventory; AUDIT = Alcohol Use Disorders Identification Test.

**Table 3:** Sleep and Health Outcomes.

Outcome	24/48: Baseline	48/96: 3-Months Post	48/96: 6-Months Post
TST	382 min (48)	406 min (39)	399 min (35)
SE	85% (7)	89% (4)	88% (3)
SOL	8.7 min (7.5)	4.9 min (2.5)	4.1 min (2.7)
WASO	58 min (24)	45 min (16)	49 min (15)
ISI	7.4 (4.2)	6.5 (4.2)	5.5 (3.5)
MAF	18.3 (8.1)	15.9 (7.7)	16.3 (6.1)
BDI-II	5.3 (4.8)	3.9 (4.6)	3.3 (4.4)
BIA	2.5 (2.7)	2.2 (2.7)	2.0 (2.8)
AUDIT	3.8 (3.7)	3.9 (3.5)	4.0 (3.5)

Mean scores reported; (STD); TST = Total Sleep Time; SE = Sleep Efficiency; SOL = Sleep Onset Latency; WASO = Wake After Sleep Onset; ISI = Insomnia Severity Index; MAF = Multidimensional Assessment of Fatigue; BDI-II = Beck Depression Inventory-II; BAI = Beck Anxiety Inventory; AUDIT = Alcohol Use Disorders Identification Test.

## Data Availability

The data presented in this study are available on request from the corresponding author due to privacy and confidentiality considerations. The data are not publicly available because of the small sample size and the potential risk of re-identification.
